# Daily data of Global Vertical Insolation in the four cardinal orientations in Burgos, Spain

**DOI:** 10.1016/j.dib.2018.11.099

**Published:** 2018-11-24

**Authors:** M. Diez-Mediavilla, C. Rodríguez-Amigo, M.I. Dieste-Velasco, T. García-Calderón, C. Alonso-Tristán

**Affiliations:** Research Group Solar and Wind Feasibility Technologies, Universidad de Burgos, Spain

## Abstract

Daily data of Global, Diffuse and Beam Horizontal Insolation and Global Vertical (North, South, East and West orientations) insolation recorded in Burgos, Spain, are presented in this paper. Ten-minute irradiance data sets are collected over forty-five months in the experimental campaign to produce estimates of daily insolation levels. This data was derived in association with the article titled: “The PV Potential of Vertical Façades: a classic approach using experimental data from Burgos, Spain” (Díez-Mediavilla et al., in press) [1]. This dataset can be used to develop and test new solar radiation and daylight models and estimate the thermal load and lighting needs in buildings for the improvement of energy efficiency.

**Specifications table**

**Table:** 

Subject area	*Environmental Sciences*
More specific subject area	*Solar Energy*
Type of data	*Excel Table*
How data was acquired	*pyranometers (Hukseflux/SR11) and pyrheliometer (Hukseflux/DR01). CR3000 datalogger*
Data format	*Filtered, cumulative daily values*
Experimental factors	*The experimental meteorological and radiometric facility is described in*[1]
Experimental features	*Cumulative hourly values have been calculated from the 10-minutes irradiance dataset using the trapezoidal rule integration. Cumulative daily values has been calculated from the hourly values.*
Data source location	*Burgos, Spain.* 42°21′04″N; 3°41′20″O; 856 m above mean sea level
Data accessibility	*All data are in this paper*
Related research article	*M. Díez-Mediavilla; M.C. Rodríguez-Amigo; M.I. Dieste-Velasco; T. García-Calderón, C. Alonso-Tristán. “The PV Potential of Vertical Façades: a classic approach using experimental data from Burgos, Spain".*[1]

**Value of the data**

• These data allow better estimates of the thermal load and the lighting needs in buildings, for the improvement of energy efficiency.
• These data can be used to develop and test new decomposition models for solar radiation and daylighting.
• The dataset can be used to calculated the total amount of solar energy incident on vertical and horizontal surfaces with different orientations.

## 1 Data

Cumulative daily data of Global (GHI), Beam (BHI), Diffuse (DHI) Horizontal Irradiation and Global Vertical Irradiation (GVI), North, South, East and West orientations, measured from January, 2014 to September 2017, in Burgos, Spain, are presented in this paper. Data are listed in the file GVI_BURGOS.xlsx. A total amount of 1272 daily data of GHI, BHI, DHI, 4 GVI is presented in this paper.

## 2 Experimental design, materials, and methods

The meteorological and radiometric facility of the Research Group SWIFT (Solar and Wind Feasibility Technologies) has provided the experimental irradiance dataset used to calculate the cumulative daily insolation data. The experimental facility is located on the roof of the Higher Polytechnic School building at Burgos University (42°21′04″N; 3°41′20″O; 856 m above mean sea level) and it is equipped with seven pyranometers (Hukseflux/SR11) and a pyrheliometer (Hukseflux/DR01) for horizontal, tilted and for each cardinal orientation vertical measurements of the irradiance values (W/m^2^). The experimental thirty-second irradiance values were recorded every 10 min and were properly analyzed and filtered using traditional quality criteria [2]. Cumulative hourly insolation values (Wh/m^2^) were calculated from the 10-min data sets series using the trapezoidal rule integration formula. Cumulative daily values were calculated from the hourly data.
